# Gemcitabine-Induced Thrombotic Microangiopathy in a Patient With Metastatic Pancreatic Cancer: A Case Report

**DOI:** 10.7759/cureus.95175

**Published:** 2025-10-22

**Authors:** Jacqueline Young

**Affiliations:** 1 Internal and Hospital Medicine, Moffitt Cancer Center, Tampa, USA

**Keywords:** eculizumab, gemcitabine-induced thrombotic microangiopathy, pancreatic cancer, ravulizumab, thrombotic micro-angiopathy

## Abstract

Thrombotic microangiopathies (TMA) are potentially life-threatening conditions caused by small-vessel platelet thrombi. Gemcitabine has been reported to induce TMA. Early detection and treatment of gemcitabine-induced TMA (GiTMA) are important to improve overall mortality. This case details a 65-year-old female with metastatic pancreatic acinar cell carcinoma who was treated with gemcitabine. The patient presented with acute kidney injury, hemolytic anemia (requiring transfusion), and thrombocytopenia. She underwent a renal biopsy, which was consistent with thrombotic microangiopathy in the subacute to chronic phase. The patient was initially treated with eculizumab, followed by a transition to ravulizumab due to the latter's less frequent dosing. Eculizumab and ravulizumab are both monoclonal antibodies targeting complement protein C5, subsequently inhibiting the terminal complement cascade and reducing inflammation and tissue injury. This case highlights the importance of recognizing GiTMA and discusses the use of complement inhibitors in its management.

## Introduction

Thrombotic microangiopathy (TMA) is characterized by a triad of thrombocytopenia, microangiopathic hemolytic anemia, and organ dysfunction, most notably acute kidney injury. TMA is a broader term encompassing conditions like hemolytic uremic syndrome (HUS) and thrombotic thrombocytopenic purpura (TTP). HUS is often linked to kidney injury, while TTP is characterized by neurological involvement. Primary TMA is considered when the cause is unknown (idiopathic) and there are intrinsic abnormalities such as ADAMTS13 deficiency, complement dysregulation, or Shiga toxin-mediated injury [1]. Secondary TMA arises due to several conditions, such as infection, autoimmune disorders, pregnancy, malignancy, or medications. Drug-induced TMA (DITMA) should be suspected when TMA develops acutely after starting a new drug [2].

DITMA is challenging to diagnose, given that laboratory tests to identify a drug etiology may not be available. DITMA occurs through either immune-mediated mechanisms or direct toxicity of the drug. Gemcitabine is a nucleoside analog that is used to treat a variety of cancers, including lymphoma, lung, bladder, breast, and pancreatic cancer. Gemcitabine has been associated with TMA in 0.02-2% of treated patients [3]. Early recognition is crucial, as mortality rates can range from 50 to 90% [3,4]. This case discusses the presentation, diagnosis, and management of gemcitabine-induced thrombotic microangiopathy (GiTMA), emphasizing the critical importance of early recognition and interdisciplinary management. It also highlights the increasing role of complement inhibition therapy in improving outcomes for this rare and life-threatening complication.

## Case presentation

A 65-year-old female with a past medical history of hypothyroidism, hyperlipidemia, and metastatic pancreatic acinar cell carcinoma with hepatic and retroperitoneal lymph node involvement presented as a referral from her primary care physician for worsening hypertension and kidney function. At her primary care office, she was noted to have a blood pressure of 190/90 mmHg and was placed on clonidine. Her creatinine was notable at 2.34 mg/dL (her baseline was around 0.8 mg/dL), and given that she also had significant lower extremity edema and fluid overload, the patient was admitted to our facility.

Regarding her oncological history, she presented three years earlier at an outside hospital for persistent abdominal pain radiating to the back. A CT abdomen and pelvis revealed a 9 cm mass in the perigastric region. Fine needle aspiration of the perigastric mass revealed a malignant epithelial neoplasm. The patient had a subsequent liver biopsy, which confirmed pancreatic acinar cell carcinoma. One month after diagnosis, the patient was started on fluorouracil, oxaliplatin, folinic acid, and irinotecan (FOLFIRINOX). Four months later, oxaliplatin was discontinued due to infusion reactions and neuropathy. One year after diagnosis, repeat imaging showed progression of disease, and the patient was started on gemcitabine and nab-paclitaxel. After nine cycles of gemcitabine and nab-paclitaxel, the patient was referred to radiation oncology for consolidative radiation therapy, given her low metastatic burden and biologically responsive disease. She received a total of five fractions of radiation therapy to the pancreatic mass. After completing radiation therapy, the patient was continued on gemcitabine and nab-paclitaxel. Repeat imaging continued to show stable pancreatic mass and stable retroperitoneal adenopathy with no new disease. The patient remained on gemcitabine and nab-paclitaxel until day one of the current hospital course.

Upon admission, the patient presented with bilateral lower extremity edema, resting dyspnea, paroxysmal nocturnal dyspnea, and oliguria. She denied any fever or neurological symptoms. Her initial vitals showed a blood pressure of 158/88 mmHg, a pulse of 93 bpm, a temperature of 98.6°F, a respiratory rate of 20/min, and oxygen saturation of 93% on room air. The initial blood test results are outlined in Table 1.

**Table 1:** Blood tests at presentation. HCO3: hydrogen carbonate, BUN: Blood urea nitrogen

Blood Test	Result	Range
Sodium	134 mmol/L	136-145 mmol/L
Potassium	5.0 mmol/L	3.5-5.1 mmol/L
Chloride	104 mmol/L	98-107 mmol/L
HCO3	20 mmol/L	22-29 mmol/L
BUN	46 mg/dL	6-20 mg/dL
Creatinine	2.50 mg/dL	0.7-1.2 mg/dL
White Blood Cell Count	11.36 k/uL	4.00-10.90 k/uL
Hemoglobin	6.9 g/dL	13.5-17.5 g/dL
Platelet Count	95,000/μL	150,000-450,000 /μL

As seen in Table 1, the patient’s creatinine was 2.5 mg/dL on admission, with her normal baseline creatinine around 0.8 mg/dL. Her initial urinalysis was significant for proteinuria (170 mg/dL) and microscopic hematuria. Her urine protein-to-creatinine ratio was 4038.0 mg/g. A renal ultrasound was obtained, which showed no developing hydronephrosis and stable mild fullness of the left intrarenal collecting system. She was evaluated by nephrology, and a workup was initiated for nephrotic syndrome and concern for gemcitabine-induced thrombotic microangiopathy (GiTMA).

Given the low hemoglobin, the patient was transfused one unit of packed red blood cells. A hemolysis panel was obtained, which found an elevated lactate dehydrogenase at 525 U/L, a decreased haptoglobin at <8 mg/dL, and an elevated reticulocyte count of 7.65%. An ADAMTS13 level was obtained and resulting in 40% (with normal being greater than or equal to 61%). This further supported a diagnosis of TMA rather than thrombotic thrombocytopenic purpura (TTP), given that ADAMTS13 activity in TTP is usually less than 10%.

An extensive diagnostic workup was performed during hospitalization to evaluate infectious and autoimmune causes. Testing for *Mycoplasma*, *Bordetella*, *parainfluenza*, *Chlamydia pneumoniae*, RSV, and COVID-19 was negative. Blood cultures remained sterile. Serologic studies for hepatitis B and C, antinuclear antibodies (ANA), antineutrophil cytoplasmic antibodies (ANCA), cryoglobulins, and lupus anticoagulant were all negative. Her complement C3 and 4 levels were within normal limits. Shiga toxin testing was also negative, effectively ruling out *Escherichia coli*-associated hemolytic uremic syndrome (eHUS).

**Figure 1:**
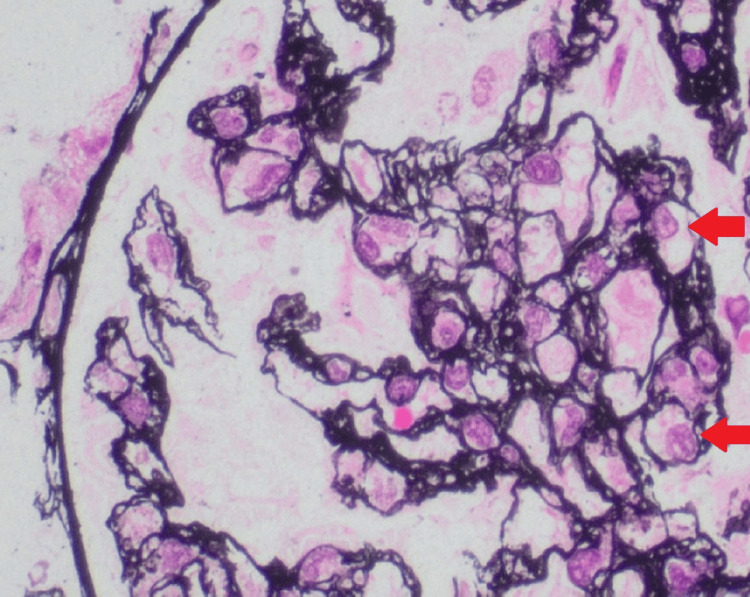
Silver stain demonstrates a glomerulus with prominent endothelial cell swelling (red arrow), which is commonly seen in conditions such as TMA and HUS. This indicates acute endothelial injury and dysfunction. TMA: Thrombotic microangiopathies, HUS: hemolytic uremic syndrome

**Figure 2:**
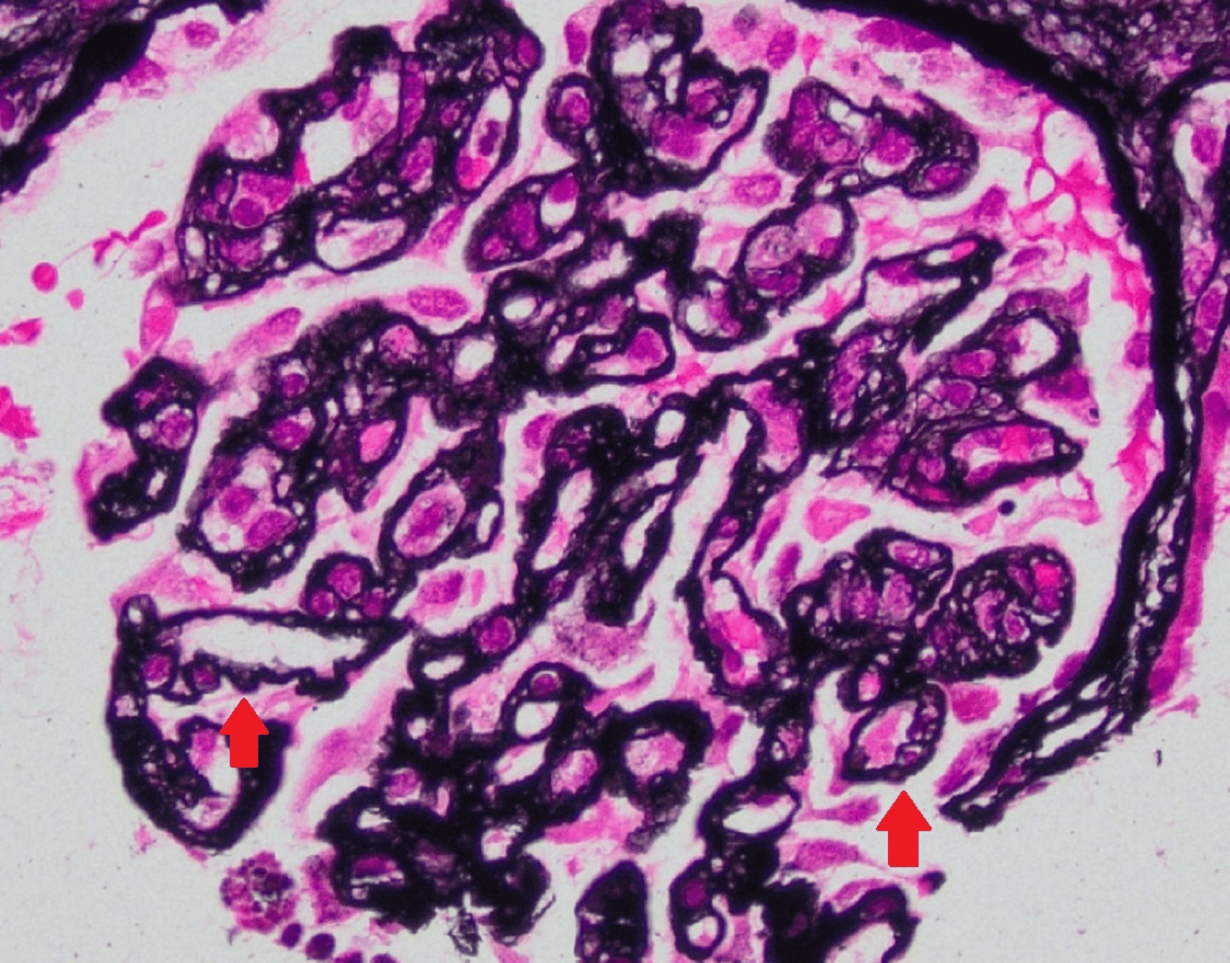
Silver stain reveals glomerular basement membrane wrinkling and duplication (red arrow). This indicates chronic or ongoing injury to the glomerular wall.

Due to the risk of progressive kidney injury, the patient was treated empirically with eculizumab alongside corticosteroids. Vaccinations against *pneumococcus* and *meningococcus* were administered prior to therapy, and penicillin V prophylaxis was initiated. Despite therapy, her kidney function declined, and she had worsening uremia, with a creatinine peaking at 4.0 mg/dL and a BUN of 106 mg/dL. This necessitated tunneled catheter placement and hemodialysis initiation. A renal biopsy was obtained and confirmed thrombotic microangiopathy in the subacute to chronic phase, with features such as endothelial cell swelling and glomerular basement membrane duplication, as seen in Figures 1, 2.

Reassuringly, the patient remained transfusion independent after starting eculizumab. She was eventually discharged while remaining hemodialysis dependent. The patient was also discharged with prednisone 60 mg daily in addition to continuing the eculizumab 900 mg IV weekly.

The patient followed up with her oncologist and nephrologist. Her oncologist transitioned her chemotherapy regimen from gemcitabine and nab-paclitaxel to 5-fluorouracil and irinotecan liposome injection, as gemcitabine was no longer a viable option in her treatment plan. After four doses of eculizumab, she had some improvement in urine output but remained dialysis-dependent. Due to the advantage of less frequent dosing, her nephrologist replaced eculizumab with ravulizumab. After switching to ravulizumab, the patient had further improvement in urine output but continued to require hemodialysis. The patient’s hemoglobin and platelet counts continued to improve.

## Discussion

Gemcitabine-induced TMA is associated with a constellation of symptoms. In a retrospective French cohort study, 97.4% of patients had AKI, 95.6% had hemolytic anemia, 74.6% had thrombocytopenia, 62.2% had hypertension, 56.7% had edema, 34.2% had proteinuria, and 22.8% had hematuria [5]. Our patient had all of the above symptoms. In addition, although the ADAMTS13 activity was decreased, the absence of fever and neurological symptoms made TTP less likely. Confirmation of the diagnosis was obtained via a renal biopsy, which was consistent with thrombotic microangiopathy in the subacute to chronic phase.

Gemcitabine is a nucleoside analog that incorporates into newly synthesized DNA, disrupting DNA replication and ultimately leading to cell death. Although the exact pathophysiology of GiTMA remains unclear, it is thought to involve endothelial cell injury, particularly within the renal vasculature [4]. Management of GiTMA begins with the prompt discontinuation of gemcitabine. Concurrent supportive care (including corticosteroids, blood transfusions, and hemodialysis) is essential to address complications. Eculizumab and ravulizumab are both monoclonal antibodies targeting the complement protein C5. They both inhibit the terminal complement cascade, reducing inflammation and tissue injury. While there is no definitive therapy for GiTMA, multiple case reports have documented successful outcomes with eculizumab, which is already approved for complement-mediated TMA [3].

Eculizumab has been the standard of care for treating atypical hemolytic uremic syndrome (aHUS) since it was approved in the United States in 2011. It requires maintenance dosing every two weeks for patients weighing 10 kg or more. Ravulizumab was approved in 2019 for the same indication. It has a longer half-life and requires less frequent dosing every 4 to 8 weeks, depending on body weight. Clinical studies have demonstrated that ravulizumab has comparable efficacy to eculizumab. In comparative studies, ravulizumab was associated with improved quality of life for both patients and caregivers and increased adherence and compliance to treatment, and was identified as the preferred treatment overall [6,7].

## Conclusions

A high index of suspicion for GiTMA is essential, particularly as the use of gemcitabine continues to increase in oncology. Given the associated high mortality, early recognition and prompt discontinuation of gemcitabine are critical. Supportive care should be initiated immediately, and treatment with a complement inhibitor such as eculizumab should be strongly considered. Ravulizumab can also be considered, given it has shown similar efficacy and improved quality of life due to the need for less frequent dosing.

In our case, the patient was empirically treated with eculizumab early in the hospital course due to the high clinical suspicion for GiTMA and the potential consequences of delayed intervention. A subsequent renal biopsy confirmed the diagnosis. This case underscores the importance of early recognition and intervention, as the patient became transfusion-independent, progressed from anuria to oliguria, and demonstrated improvement in both platelet count and hemoglobin levels. However, it is important to note that the patient remained dialysis-dependent, which limits the assessment of the treatment efficacy. This outcome may be attributed to subacute to chronic changes already seen on the renal pathology.
